# Growth dynamics of Rathke's Cleft cyst: a risk score system for surgical decision making

**DOI:** 10.1007/s00701-024-06299-1

**Published:** 2024-10-15

**Authors:** Mohammad Bilal Alsavaf, Jaskaran S. Gosal, Kyle C. Wu, Shoban Babu Varthya, Moataz D. Abouammo, Luciano M. Prevedello, Ricardo L. Carrau, Daniel M. Prevedello

**Affiliations:** 1 Department of Otolaryngology-Head and Neck Surgery, The Ohio State University Wexner Medical Center, 410 W 10Th Ave, Columbus, Ohio 43210-1228 USA; 2 Department of Neurological Surgery, The Ohio State University Wexner Medical Center, 410 W 10Th Ave, Columbus, Ohio 43210-1228 USA; 3 Department of Neurosurgery, All India Institute of Medical Sciences (AIIMS), Jodhpur, Rajasthan India 342005; 4 The James Cancer Hospital and Solove Research Institute, The Ohio State University, 410 W 10Th Ave , Columbus, Ohio 43210-1228 USA; 5 Department of Pharmacology, AIIMS Jodhpur, Jodhpur, India; 6 Department of Otolaryngology-Head and Neck Surgery, Tanta University, Tanta, Egypt; 7 Department of Neuroradiology, Wexner Medical Center at The Ohio State University, Columbus, OH USA

**Keywords:** Rathke cleft cyst, Growth rate, Risk score, Smoking, Pituitary dysfunction, Prognosis, Early surgery, Imaging, Predictive model, Pituitary function, Cerebrospinal fluid leak, Skull base, Endoscopic endonasal approach

## Abstract

**Objective:**

Rathke's cleft cysts (RCCs) exhibit variable growth patterns, thus posing a challenge in predicting progression. While some RCCs may not cause symptoms, others can insidiously cause pituitary dysfunction, which is often irreversible, even following surgery. Hence, it is crucial to identify asymptomatic RCCs that grow rapidly and pose a higher risk of causing endocrinologic dysfunction. This enables timely surgical intervention to prevent permanent damage. Our study examines the growth rate of RCCs, identifies factors that accelerate growth, and discusses the clinical implications of these findings.

**Methods:**

A retrospective analysis of a prospectively maintained electronic database revealed 45 patients aged 18–80 years who underwent endoscopic endonasal surgery (EEA) for RCCs between 2010 and 2022 at our center. Of these, 20 required *early* operative intervention. The remaining 25 patients were followed closely clinically and radiologically before requiring surgery (*initial conservative* management group). We conducted an analysis of the factors predicting growth over time in this group. Using a regression model, we constructed a risk score system to predict RCC growth over time.

**Results:**

Patients in the *initial conservative* group had smaller cysts and were generally older than those in the *early surgery* group. Patients with preoperative pituitary dysfunction showed a higher median growth of 1.0 mm in the longest diameter compared to those with normal pituitary function, with an increase of 0.5 mm. A sum of annual cyst growth of all (z, y, x) diameters, at a rate of 3 mm or greater, was associated with a clinically significant increase in the risk of pituitary dysfunction, exceeding 50%.The most significant factors predicting rapid growth in RCCs were smoking status, age, and T1-weighted magnetic resonance imaging (MRI) intensity of cysts. Smoking was the most critical risk factor for rapid cyst growth (p =  < .001). Our risk score system accurately predicted RCC growth with a 74% accuracy rate, 73% sensitivity, and 75% specificity.

**Conclusion:**

Our analysis showed a strong link between active smoking and the rapid growth of RCC. This novel finding has significant preventive implications but needs validation by a large population database. Surgical intervention for RCC currently is often reserved for symptomatic cases. However, utilizing our risk-based scoring system to predict rapidly growing cysts may indicate early surgery in minimally symptomatic patients, thereby potentially preserving pituitary function.

## Introduction

Rathke's cleft cysts (RCCs) are benign sella-suprasellar lesions arising embryologically from Rathke’s pouch remnants [16]. They constitute only 1.8% of pituitary and sellar tumors; however, autopsy studies reveal incidental RCCs in up to 1/3 of the population [4, 10, 11, 14, 15, 20, 27]. Most RCCs are discovered incidentally and do not produce symptoms, but these cysts can grow and become symptomatic.

Currently, the standard treatment for symptomatic RCCs is surgical intervention, while conservative follow-up is recommended for asymptomatic RCCs [4]. However, a conservative approach may put patients at risk of developing irreversible endocrinologic dysfunction in rapidly progressing RCCs. With the exception of hyperprolactinemia, pre-existing pituitary dysfunction generally does not improve after surgery [9]. With recent advancements in neuroendoscopy and RCC surgery techniques, endoscopic endonasal surgical drainage and marsupialization are safe and well-tolerated treatment options [7, 35]. Identifying asymptomatic RCCs that are more likely to grow quickly and cause pituitary dysfunction is crucial to initiating timely surgical intervention to prevent permanent damage.

To our knowledge, no study has yet analyzed the growth rate of RCCs over a follow-up period in patients who have received a confirmed diagnosis. Such a study would be beneficial in identifying the high-risk asymptomatic RCCs, which tend to grow faster and cause anterior pituitary dysfunction and potential vision loss. It would then help determine the optimal timing for treatment, especially in minimally symptomatic or asymptomatic patients. Early surgery in such patients can help preserve pituitary function and avoid potential risks from delayed intervention. Additionally, it can help weigh the advantages and disadvantages of early versus delayed intervention.

In this study, we compared the characteristics of patients who were managed expectantly with those who were operated on early regarding presenting symptoms, neuroimaging findings, pathology results, and pre-and postoperative complications. Additionally, we analyzed the growth rate of preoperatively followed-up patients and developed a preliminary clinical scoring system- the RCC Risk Score (RCC-RS), to assist in clinical decision-making and establish objective surgical indications in asymptomatic/minimally asymptomatic patients.

## Methods

### Patient Population

We retrospectively analyzed the prospective electronic database for RCCs at The Ohio State University Wexner Medical Center between 2010 and 2022. The study was performed according to the STROBE guidelines [30]. All patients met the criteria for retrospective chart review according to Institutional Review Board (IRB) guidelines (2022C0029). Forty-five consecutive patients between the ages of 18 and 80, with this diagnosis based on final histopathological analysis, were included in the study. Patients with suspected RCC who did not undergo surgical resection were excluded as a control group due to uncertain histopathological confirmation. The size of the RCCs was primarily obtained from radiographic reports based on the patients' pre- and post-operative magnetic resonance imaging (MRI), as calculated by a board-certified neuroradiologist. When reports were missing or incomplete, sizes were calculated from images using NilRead Diagnostic Image Viewer (Hyland, Ohio). Endocrine status was evaluated using biochemical laboratory data or endocrinology office visits. Patients who had RCC surgery outside our hospital or were treated with a non-endoscopic endonasal transsphenoidal approach (EETA) were excluded from the study.

### Management strategy in two patient groups

We divided the patient data into two arms according to the initial management strategy offered to RCC patients. The asymptomatic patients (whose cysts were discovered incidentally) or those with minor, manageable symptoms were closely observed. They were operated on later if their cysts became symptomatic or with symptoms that were interfering with the patient’s lifestyle (for example, medications for headaches, fertility, energy level, etc.). On the other hand, symptomatic RCCs were immediately offered surgery at presentation. Patients in both groups were operated on by the standard endoscopic endonasal transsphenoidal approach described in an earlier report [9].

### Pathological Tissue Analysis

During surgery, tissue samples were taken from the cyst wall for preliminary and final analysis. The samples were initially frozen for preliminary diagnosis, with the remainder being permanently stored for final analysis.

### Statistical Analysis

Statistical analyses were conducted using R statistical software (version 4.1.1). Means and standard deviations in the sample are presented for approximately normally distributed variables. For categorical variables, the number and percentage of patients in the sample are shown for each level of the variable. Univariate associations between outcomes and the independent variables of interest were tested using two-sample t-tests for continuous variables (the size of the cyst and the patient's age) and Fisher’s exact test for dichotomous or categorical variables (all other variables). The risk score system was developed using regression modeling techniques. The outcome variable of interest was the growth rate of the cyst, measured either as the sum of three dimensions (cubic units per year) or the diameter (units per year). Multiple linear regression models were fitted to predict the outcome variable using predictor variables such as age, smoking status (current, former, or never), MRI T1 and T2 values, and their interactions with time. Model selection was performed to identify the best set of predictors, and the final model for predicting cyst diameter growth rate included age, smoking status, and MRI T2 value. A 5% significance level was used to determine statistical significance for all tests.

### Risk Score Model

The risk score formula was derived as a linear combination of the selected predictors, with coefficients estimated from the regression model. Cut-off values for the risk score were determined to classify patients into low-, medium-, or high-risk categories based on the distribution of scores in the sample. The performance of the risk score model was evaluated using sensitivity and specificity estimates, calculated from the model's ability to correctly classify patients into surgical decision categories (immediate surgery or conservative approach). Receiver operating characteristic (ROC) curves and the area under the curve (AUC) metric were also utilized to assess the overall discriminative ability of the risk score model.

## Results

### Demographics and Clinical Presentation of Patients

In the initially conservative treatment group (n = 25), the demographic characteristics were comparable to those in the immediate treatment group (n = 20). The mean age of patients in the conservative treatment group was 41.88 ± 20, and 72% were female. The average follow-up period before surgery was 59 ± 44.8 months. Most cysts were located in the sella-suprasellar region, followed by the sella, and were least common in the suprasellar area alone (60%, 36%, and 4%, respectively). Hyperintense T1 (73%) and hypointense T2 (51%) were the most common features on MRI images. At time of surgery, there was no significant difference in the presenting symptoms between the early treatment and conservative group. The most frequently reported symptoms for the latter group included headache (68%) and visual impairment (44%), followed by dizziness (16%), seizure (8%), nausea and vomiting (4%), fatigue (4%), and dysmenorrhea (4%). (Table 1.)

**Table 1:** Comparison of clinical characteristics between immediately treated RCC and conservatively treated RCC

		Mean Immediate	SD Immediate	Mean Conservative	SD Conservative		
Age		36.45	19.96438	41.88	19.90251		
Size		16.335	4.737563	13.88	5.041577		
		20# Immediate	% Immediate	25# Conservative	% Conservative	% Difference	P-value
Gender	Female	15	75	18	72	3	1
Location	Sella	4	20	9	36	-16	0.579881
	Suprasellar	1	5	1	4	1	NA
	Sella-Suprasellar	15	75	15	60	15	NA
Smoking	Former	0	0	9	36	-36	0.003496
	Current	4	20	6	24	-4	NA
	Never	16	80	10	40	40	NA
MRI1	Hyperintense	14	70	19	76	-6	0.484913
	Hypointense	5	25	3	12	13	NA
	Isointense	1	5	3	12	-7	NA
MRI2	Hyperintense	4	20	8	32	-12	0.281715
	Hypointense	9	45	14	56	-11	NA
	Isointense	1	5	1	4	1	NA
	Mixed-intensity signals	6	30	2	8	22	NA
Presenting Symptoms	Visual Impairment	9	45	11	44	1	1
	Headache	16	80	17	68	12	0.502185
	Dizziness	2	10	4	16	-6	0.678087
	Nausea and Vomiting	3	15	1	4	11	0.308702
	Weight gain	1	5	1	4	1	1
	Fatigue	2	10	1	4	6	0.577167
	Dysmenorrhea	0	0	1	4	-4	1
	Seizures	0	0	2	8	-8	0.494949
Hypopituitary Function		7	35	8	32	3	1
Signs of Inflammation on Histopathology		4	20	6	24	-4	1
Intraoperative CSF Leakage		6	30	4	16	14	0.301052
Recurrence		4	20	3	12	8	0.682214

### Cyst growth rate and pituitary dysfunction in conservative group

Cysts in patients with existing pituitary dysfunction grew faster over time compared to patients without pituitary dysfunction (p = 0.02). In particular, the cyst in a patient with pituitary hypofunction grew 0.65 mm larger after one year compared to a similar patient without decreased function.

The median growth across all dimensions was found to be 2.1 mm per year. Among patients with pituitary dysfunction, the median growth was again higher at 2.9 mm per year compared to those without pituitary dysfunction (median increase of 1.7 mm annually).

### Rate of cyst growth and the influencing factors in conservative group

1. Effect of smoking

During the initial visit, non-smokers (10 patients) and former smokers (8 patients) tended to have smaller estimated cyst dimensions (longest diameter) and volume than active smokers (7 patients), with differences of -3.54 mm -1441.62 mm^3^ and -0.78 mm -646.76 mm^3^, respectively. Nevertheless, these differences were not statistically significant, with p-values of 0.12 and 0.2 and p = 0.74 and 0.57, respectively.

However, on follow-up, the longest diameter and volume of the RCC in active smokers increased more rapidly over time compared to non-smokers (p = 0.002 and < 0.001, respectively) and former smokers (p = 0.01 and < 0.001, respectively).

2 Effect of time, age, and gender

Growth of the RCC is correlated to age and gender, occurring more rapidly in younger (age < 35 vs > 35) (p = 0.03) and male patients (p = 0.048). For example, the median diameter of the cyst grew 0.47 mm after one year in 65-year-olds compared to 0.68 mm in 35-year-olds, and after two years, 1.53 mm growth was observed in 65-year-olds compared to 1.74 mm larger in 35-year-olds. For current smokers, cysts are expected to increase in size over time, but the growth rate is slower for older patients.

3 Effect of cyst location

Sellar-suprasellar located cysts are expected to be significantly larger than those in other locations (suprasellar and sellar) (p = 0.002). The mean difference in cyst size between these types of patients is not expected to change over time.

4 Effect of T1MRI

Those with hypointense T1-weighted MRI were found to have a larger cyst (in diameter) at presentation than those with hyperintense T1 MR (the estimated difference is 6.6 mm, p = 0.01). Also, they grew notably faster (0.65 mm) compared to hyperintense cysts (p = 0.016). At the time of diagnosis, the diameter and volume of cysts in patients with intermediate T1-weighted MR were similar to those in patients with hyperintense MRI T1 (p = 0.59 and p = 0.81, respectively). However, cysts with intermediate T1-weighted MRI grew more slowly than those with hyperintense T1-weighted MR. Specifically, the cyst’s diameter and volume with hyperintense T1-weighted MR grew significantly faster per year (1.07 mm, 339.42 mm^3^) than those with intermediate T1-MR (p = 0.000046, p = 0.008, respectively).

5 Effect of MRI T2

**Fig. 1:**
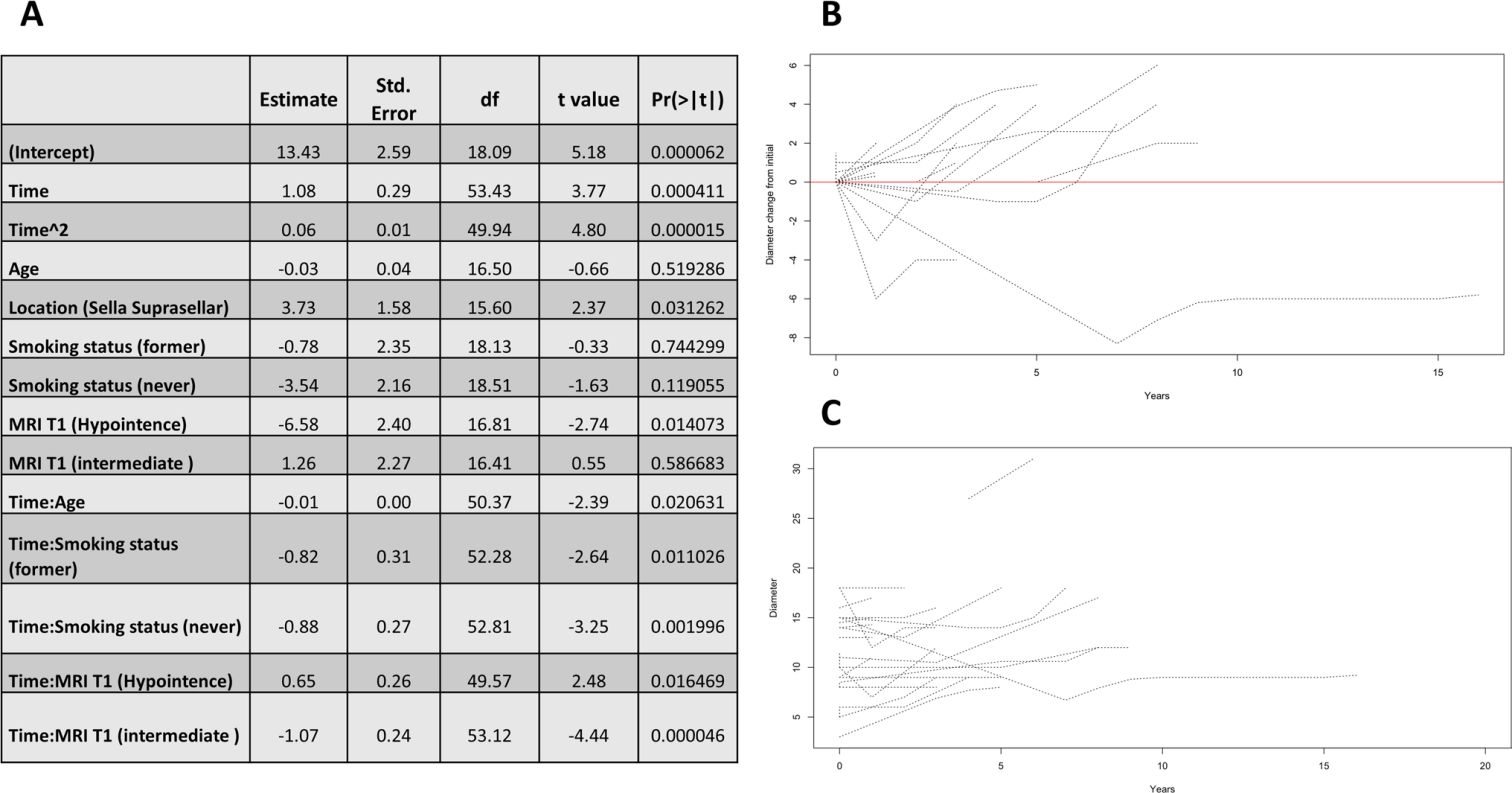
**A**. Illustrates the estimated effect of factors on the growth of the first dimension only (e.g., the outcome is 'a' if the size is "a x b x c"); **B**. Shows the growth of the first dimension over time from the point of zero since the detection of RCC; **C**. Shows the growth over time from the actual size at the time of first presentation

**Fig. 2:**
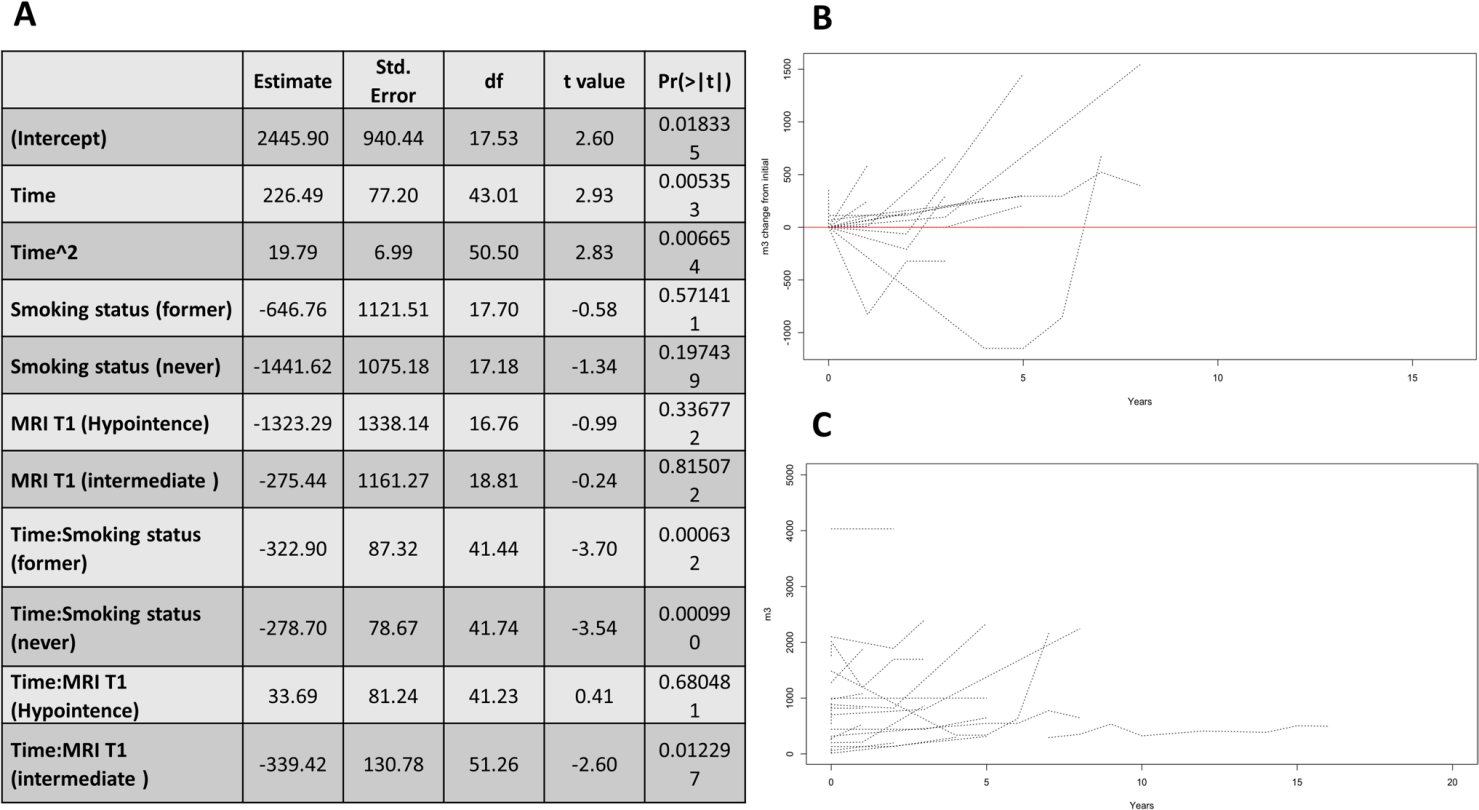
**A**. Illustrates the estimated effect of factors on the growth of the whole size only (e.g., the outcome is 'a' if the size is "a x b x c"). **B**. Shows the growth of the size over time from the point of zero since the RCC detection. **C**. Shows the growth of the size over time from the real size at first presentation

Patients with mixed-intensity T2-weighted MR had a significantly smaller cyst diameter than others, but the growth rate over time did not depend on T2-weighted MRI. (Figs. 1 and 2).

## Risk score model

**Fig. 3:**
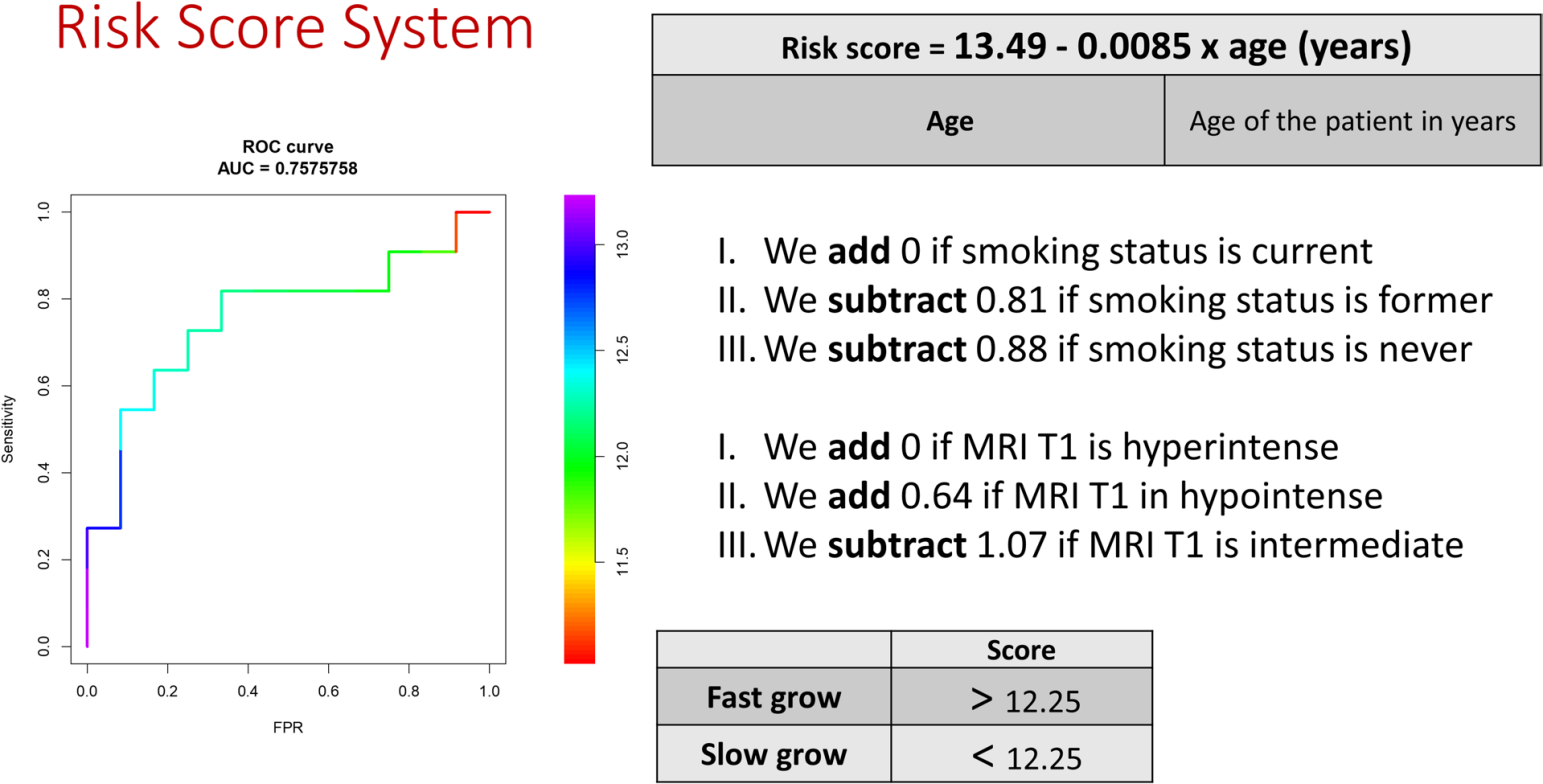
Illustrates the risk score system that uses age, smoking status, and MR T1 instantaneities as input factors in the equation. If the score is greater than 12.25, it indicates that the patient may have fast-growing RCC, and surgery is recommended. If the score is less than 12.25, observation is recommended

The risk score model accurately predicts patient outcomes in terms of faster versus slower growth compared to the median growth rate in our sample. The model demonstrates a 74% accuracy rate with 73% sensitivity and 75% specificity **(**Figs. 3**).** The outcome of interest in this study was defined as growth greater than 0.5 mm per year, which is considered "faster growth" based on the median growth rate of the sample. Using this model, we estimated a risk score for each patient and classified those with a score greater than 12.25 as having "faster growth" and those with a score less than 12.25 as having "slower growth".

## Discussion

The present study offers novel insights into the dynamic changes in RCC size, identifies the risk factors associated with rapid growth, and highlights the clinical implications of these findings. Our findings substantiate that patients exhibiting a growth rate above average are predisposed to an elevated risk of pituitary dysfunction, where over half of these patients were presented with pituitary dysfunction. Consequently, we have developed a reliable risk score system to identify individuals most susceptible to this condition. Implementing this scoring system in clinical practice could provide timely surgical intervention and avert the onset of irreversible pituitary dysfunction.

### Literature summary on conservative management vs early surgery

The literature has considerable controversy regarding the optimal management of RCC patients with large cysts but minimal or no symptoms [7]. In prior research, a notable constraint in the conservative group was the selection criteria based on imaging features without histopathological confirmation. Notably, T1 and T2 weighted MRI intensity exhibited significant variability, with typical imaging features present in less than half of the patients in one study [23]. This variability can lead to diagnostic confusion with other sella-suprasellar lesions, including craniopharyngiomas, chondroid tumors, abscesses, mucoceles, and lipomas [17, 25]. Another area of criticism in the literature is that the stability or regression of the lesion might be due to short-term regression, cyst rupture, and bleeding followed by resorption or scarring. Chronic elevation of intrasellar pressure has been proposed as a possible cause of the rupture [3, 12, 21, 22].

In our study group, we observed temporary regression exclusively in patients with hypointense T2, implying the potential validity of the bleeding resorption theory or water absorption and protein concentration [2]. Furthermore, we observed cases of stable and regressing cysts that eventually experienced rapid rebound growth after several years. Reported instances of shrinking RCC are limited in the literature [3, 12, 21, 24]. Truong et al. presented a case of RCC that entirely vanished. Still, the patient was left with persistent diabetes insipidus and thyrotropin deficiency, underlining the importance of early intervention to prevent permanent endocrinopathy [3]. Maruyama et al. observed a regression of cyst size with high-dose steroid use in a patient with secondary hypoadrenocorticism. However, the patient ultimately required surgery due to medication side effects. The authors attributed part of the retraction to the anti-inflammatory effect, although histopathology did not confirm inflammation [28].

### The importance of early surgical intervention

The management of patients with imaging suspicious for RCC with minimal or no symptoms requires informed decision-making, as early surgical intervention could mitigate the risk of intraoperative cerebrospinal fluid (CSF) leak and irreversible pituitary hypofunction. Previous reports have established a substantial correlation between increasing cyst size and the likelihood of intraoperative CSF leak [9]. Nonetheless, the surgical intervention itself presents a risk of CSF leak, with reported incidence rates ranging from 8.5% to 52.0% in the literature [1, 26, 29, 31, 34, 36].

Preoperative pituitary dysfunction has been observed in 26% to 81% of patients [5, 8, 19, 26, 32, 33], while postoperative adenohypophysis dysfunction is rare. The preoperative dysfunction is often permanent and does not resolve with surgery unless it is related to hyperprolactinemia due to the stalk effect [5, 8, 18, 26]. The mechanism underlying this pituitary hypofunction is believed to be linked to chronic compression and inflammatory effects [8, 13]. Analysis of our published data indicates that pituitary hypofunction is approximately 20 times more frequent in individuals with histological evidence of inflammation, and we observed faster growth rates in such patients (p = 0.02) [9].

Appropriate patient selection for early surgical intervention can reduce the occurrence of anterior pituitary endocrinopathies and the consequent need for hormonal replacement therapy. Notably, the thyroid and gonadal axes appear to be the most frequently affected endocrine axes in our patient population, suggesting that those axes are the most vulnerable.

### Relation of smoking with RCC growth

No report in the literature previously reported a correlation between smoking and the growth of RCCs. Our investigation is the first to reveal the association between smoking and accelerated RCC growth. The precise mechanism underlying this phenomenon, however, remains unclear. As smoking is recognized to cause chronic inflammation with irritant properties [6], we theorize there could be a link between smoking and inflammatory changes in histopathology encountered in some of the RCCs. However, the analysis of our data did not support this theory. Intriguingly, even ex-smokers exhibited a more rapid tumor expansion rate compared to non-smokers, implying a chronic rather than acute influence of smoking. It is also noteworthy that a previous report noted cyst regression with steroid usage, but no histopathological inflammatory markers were observed in that study [28].

Nevertheless, our data clearly identifies smoking as the most critical risk factor for the rapid growth of RCCs, and this novel association needs to be validated in a large patient population. Patients should be counseled to refrain from smoking to avoid a rapid increase in cyst size.

### The risk score guides asymptomatic RCC management

No clinical evidence supports any particular size or growth rate above which surgery should be offered to asymptomatic RCC patients. Hence, the risk score proposed in this study could guide surgeons in managing asymptomatic patients with large cysts. Our study suggests that the rapid growth rate of cysts and their fluid consistency might significantly contribute to pituitary dysfunction. Notably, patients with annual growth rates below a total of 3 mm in all diameters did not experience pituitary dysfunction. In contrast, an annual growth rate of 3 mm or greater was associated with a clinically significant increase in the risk of pituitary dysfunction, exceeding 50%.

Furthermore, upon analyzing the cysts that had the greatest change in the sum of all diameters from the time of initial presentation to surgery regardless of the time of follow up, we observed that most of them exhibited cysts with hypointense T2 and hyperintense T1, appearing as solid, nodule-like structures with thick material observed inside them during surgery. Although our study’s limited patient population precludes a clear correlation between cyst consistency and pituitary dysfunction, it remains a promising avenue for future research.

Understanding the factors influencing the cyst’s changing size is critical in selecting the most suitable patients for surgery. This study identifies the factors associated with a faster tendency for cyst growth, thus helping to develop a high-sensitivity and specificity scoring system based on age, smoking status, and T1 MRI intensity. The scoring system aids in identifying patients who are at greater risk of pituitary dysfunction and require earlier surgical intervention.

## Limitations

The current study presents a new strategy for managing asymptomatic RCCs with the aim of reducing preoperative pituitary dysfunction and intraoperative cerebrospinal fluid leaks. However, it is important to note the retrospective nature of this study and that the sample size of this investigation was modest, and imaging presentations of RCCs are complex and varied, requiring further analysis with larger groups of patients. We also recommend follow-up assessments that include evaluations of glandular function and imaging to fully understand the correlation between changes in RCC size and pituitary function. Future studies could validate the risk score proposed in this paper.

The exclusion of non-surgical patients due to uncertain diagnosis, while ensuring pathological confirmation of RCC, significantly limits our ability to compare outcomes with a proper control group and may introduce selection bias. Our findings relate only to the natural history of cases that ultimately require surgical resection. This approach prevents us from drawing conclusions about the natural history of RCCs in patients who never needed surgery. For example, there might be smokers whose cysts never progressed to require surgical intervention. Future studies with a non-surgical control group are needed for further validation.

## Conclusions

An annual RCC growth rate of 3 mm or above is associated with a high risk of developing pituitary dysfunction, which may not reverse with surgery. While the symptomatic presentation may serve as the principal criterion for surgical intervention in RCC, identifying faster-growing symptomatic cysts may facilitate a preemptive surgical approach, thereby mitigating the risk of permanent pituitary insufficiency. A significant positive association exists between active smoking and RCC expansion, thereby providing evidence for smoking restriction in these patients.

## Data Availability

No datasets were generated or analysed during the current study.

## Abbreviations

(AUC): Area Under the Curve
(CSF): Cerebrospinal Fluid
(EEA): Endoscopic Endonasal Approach
(EETA): Endoscopic Endonasal Transsphenoidal Approach
(IRB): Institutional Review Board
(MRI): Magnetic Resonance Imaging
(RCC): Rathke’s Cleft Cyst
(ROC): Receiver Operating Characteristic
